# Tryptophan Kynurenine Pathway-Based Imaging Agents for Brain Disorders and Oncology—From Bench to Bedside

**DOI:** 10.3390/biom15010047

**Published:** 2025-01-01

**Authors:** Erik Stauff, Wenqi Xu, Heidi H. Kecskemethy, Sigrid A. Langhans, Vinay V. R. Kandula, Lauren W. Averill, Xuyi Yue

**Affiliations:** 1 Department of Radiology, Nemours Children’s Health, Delaware, Wilmington, DE 19803, USA; erik.stauff@nemours.org (E.S.); wenqi.xu@nemours.org (W.X.); heidi.kecskemethy@nemours.org (H.H.K.); vinay.kandula@nemours.org (V.V.R.K.); lauren.averill@nemours.org (L.W.A.); 2 Diagnostic & Research PET/MR Center, Nemours Children’s Health, Delaware, Wilmington, DE 19803, USA; sigrid.langhans@nemours.org; 3 Division of Neurology, Nemours Children’s Health, Delaware, Wilmington, DE 19803, USA; 4 Department of Radiology, Thomas Jefferson University, Philadelphia, PA 19107, USA

**Keywords:** tryptophan, kynurenine pathway, radiopharmaceutical, PET, brain imaging, clinical translation

## Abstract

Tryptophan (Trp)-based radiotracers have excellent potential for imaging many different types of brain pathology because of their involvement with both the serotonergic and kynurenine (KYN) pathways. However, radiotracers specific to the kynurenine metabolism pathway are limited. In addition, historically Trp-based radiopharmaceuticals were synthesized with the short-lived isotope carbon-11. A newer generation of Trp-based imaging agents using the longer half-lived and commercially available isotopes, such as fluorine-18 and iodine-124, are being developed. The newly developed amino acid-based tracers have been demonstrated to have favorable radiochemical and imaging characteristics in pre-clinical studies. However, many barriers still exist in the clinical translation of KYN pathway-specific radiotracers.

## 1. Introduction

In our human body, approximately 90% to 95% of tryptophan (Trp) is metabolized through the unique kynurenine (KYN) pathway. The study of KYN pathway-based radiotracers has been an area of growing interest in the field of neuroradiology because of these compounds’ potential in diagnosing and monitoring the treatment response of several types of medical conditions, most notably tumors and epilepsy [1,2]. These agents are also developed to monitor disease progression and stratify diseases. Positron emission tomography (PET) has been the gold standard for molecular imaging of brain disorders for many years, and, as hybrid technologies such as PET/magnetic resonance imaging (MRI) and PET/computed tomography (CT) being refined, the quality of the modality has only improved [3]. Fludeoxyglucose-18 (FDG) is the most widely used PET imaging agent for brain pathology. However, because of the high glucose metabolism of the brain, it is often difficult to distinguish between tumor metabolism and the natural glucose metabolism in the organ from increased FDG uptake caused by pathological conditions. Trp-based tracers have the potential to overcome this limitation because of their unique metabolism through the KYN pathway, which is different from the standard glucose utilization and shows specific lesion accumulation. Brain tissue can be imaged using Trp-based PET tracers without large amounts of background glucose appearing on the scan, in contrast to FDG images with heavy glucose use in the brain tissue. For PET imaging, the fact that many tumors have higher amino acid uptake than the surrounding brain tissue presents a significant advantage in imaging tumor pathology [4]. While many amino acid-based radiopharmaceuticals have this advantage over FDG, Trp-based tracers have additional potential because of the involvement of the KYN pathway in both immune system and inflammatory responses to pathological conditions, as well as the organification of KYN metabolites. The purpose of this paper is to review the current state of research on Trp imaging of the KYN pathway in the brain and oncology and to discuss the translation of the current body of pre-clinical work to the clinical setting.

## 2. Methods

**Figure 1:**
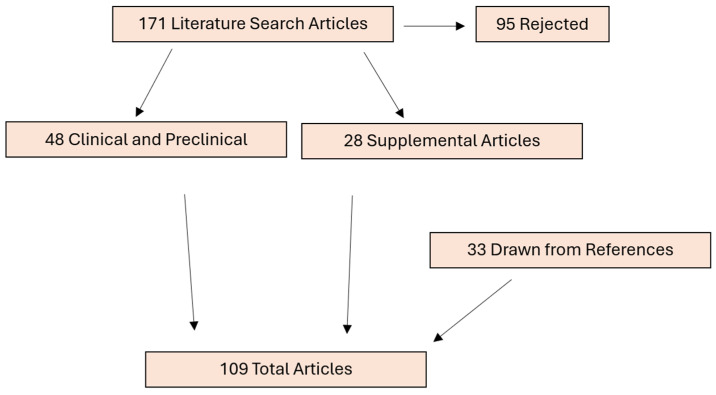
Results from literature search and included journal articles.

A literature search was performed using Covidence (Melbourne, Australia) to search multiple journal databases from January 2005 through April 2024 using the search terms “tryptophan”, kynurenine”, or “radiopharmaceutical”. The Covidence search yielded 171 peer-reviewed journal articles. These articles were screened by one reviewer for content pertaining to Trp and/or the KYN pathway, yielding 76 articles. Next, the 76 articles were reviewed for content pertaining to radiopharmaceutical imaging of the kynurenine pathway in pre-clinical or clinical conditions. Forty-eight articles included clinical or pre-clinical content, and 28 articles were identified as containing pertinent supplemental information related to KYN pathway molecular imaging. In addition, reference sections of these articles were examined for any potentially relevant content that might have been missed by the initial search. With the inclusion of seminal articles, this process yielded 33 additional articles, for a total of 109 included works (Figure 1).

## 3. Trp Metabolism

Trp is an essential amino acid that must be consumed through diet. It has a unique chemical structure among amino acids in that it has a structural indole ring [5], consisting of a benzene and a pyrrole ring, which contributes to its multiple metabolic schemes. Approximately 1% of dietary Trp is incorporated into proteins that the body builds, but much of it is catabolized through two major metabolic pathways: appropriately 90% to 95% through the immune-regulatory KYN pathway and 4% through the serotonin pathway. In addition, a small fraction of unabsorbed Trp is metabolized by the gut microbiota-derived indole pathway into indole and indole derivatives. These pathways are more specific to L-Trp and not its D-counterpart. It had been demonstrated in pre-clinical trials that the cellular uptake of the L-isomer is up to 10 times greater than that of its D-counterpart [6], with some tracers showing negligible uptake of the D-isomer altogether [7].

Trp is transported into cells by the large amino acid transport system (LAT). These LAT transporters play an important role in Trp metabolism because of their capability to import large branched and aromatic neutral amino acids across the cellular plasma membrane [8]. Four subtypes of the LAT transporter have been identified, numbered 1 to 4. LAT1 and LAT2 are most pertinent to this discussion, as they are primarily responsible for the transport of Trp across the cell membrane and play an essential role in the KYN pathway-associated cancers [9,10]. Around 80% of Trp is transported via the LAT1 transporter under low Trp concentrations (50 nM), although it has been demonstrated that LAT2, LAT3, and LAT4 ramp up their involvement in Trp transport to around 21% at high concentrations (0.5 mM) of serum Trp [11]. This kinetic variability of Trp transport adds another layer of complication to the study of Trp tracer pharmacokinetics, as the amount of Trp available for metabolism is ultimately dependent on these transporters.

**Figure 2:**
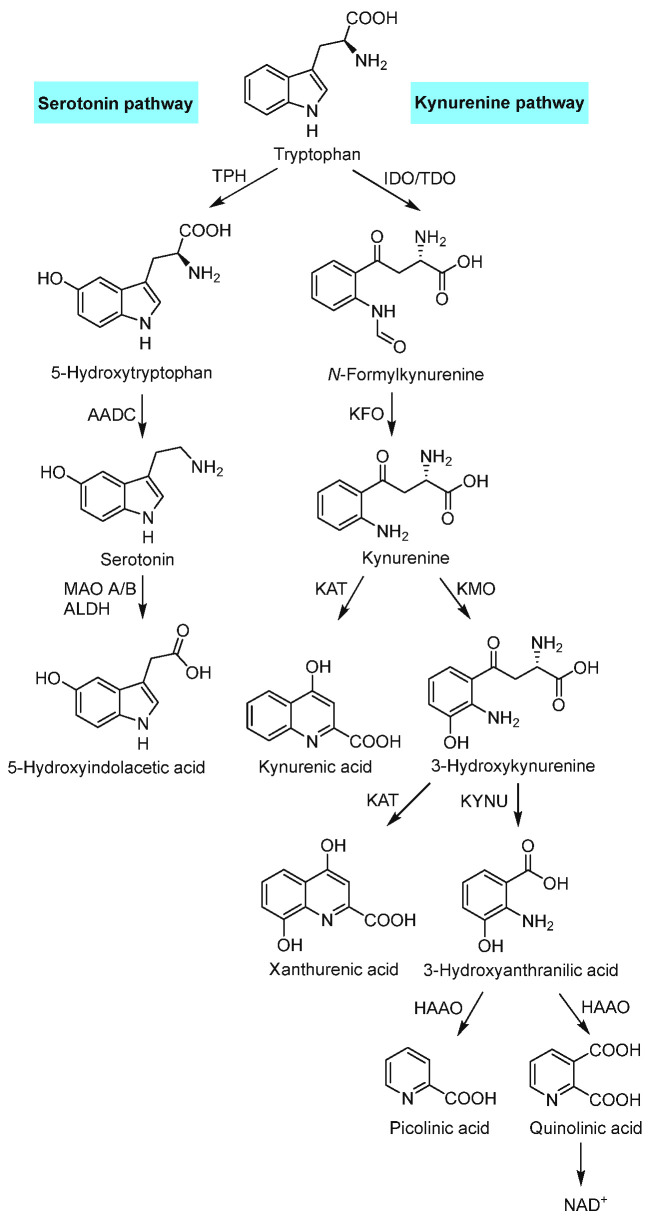
Major metabolism pathways: serotonin and kynurenine pathways. TPH, tryptophan hydroxylase; IDO, indoleamine 2,3-dioxygenase; TDO, tryptophan 2,3-dioxygenase; AADC, aromatic amino acid decarboxylase; KFO, kynurenine formylase; MAO A/B, monoamine oxidases A and B; ALDH, aldehyde dehydrogenase; KAT, kynurenine aminotransferase; KMO, kynurenine-3-monooxygenase; KYNU, L-kynurenine hydrolase; HAAO, 3-hydroxyanthranilic acid dioxygenase; NAD^+^, nicotinamide adenine dinucleotide.

One of the metabolic pathways for Trp is the serotonin pathway (Figure 2), which leaves the indole ring intact. This biosynthesis begins with 5-hydroxylation of the indole ring by the enzyme Trp hydroxylase (TPH) producing the amino acid 5-hydroxytryptophan (5-HTP). This hydroxylation, the addition of a hydroxyl (-OH) to the compound, is the rate-limiting step in the production of the neurotransmitter serotonin and occurs primarily in the gut, although a small amount (5%) of this process takes place in the central nervous system (CNS). Following this, the compound is decarboxylated by the catalyst aromatic amino acid decarboxylase (AADC) into serotonin. This neurotransmitter plays important primary roles in emotional and cognitive processes in the brain as well as support of the digestive system. Systemic serotonin is broken down by the liver using monoamine oxidases A and B (MAO A/B) as well as aldehyde dehydrogenase (ALDH) into the metabolite 5-hydroxyindolacetic acid. Some of the serotonin is also converted into melatonin by the pineal gland.

The major metabolic pathway for Trp is the KYN pathway (Figure 2). This pathway begins with the oxidation of the indole ring by one of two enzymes: tryptophan 2,3-dioxygenase (TDO) or indoleamine 2,3-dioxygenase (IDO). TDO is found primarily in the hepatic system, while IDO is widespread in many tissue types, including the CNS, specifically microglia, astrocytes and neurons [12]; and lungs, heart, kidneys, and intestines. IDO and TDO are the rate-limiting enzymes in the KYN pathway and have garnered attention because of their role in the immune system and the inflammatory response, of which regulation is controlled by the rate of Trp metabolism. Either of these two enzymes can oxidize the indole ring, rendering *N*-formylkynurenine, which is then hydrolyzed into the intermediary KYN, but TDO exists primarily outside the brain making IDO more relevant to neuroimaging.

Kynurenine then has several different synthesis pathways with multiple downstream metabolites and functions. Kynurenine aminotransferase (KAT) catalyzes the synthesis for kynurenic acid (KYNA), which carries out a large variety of functions in the human body but is mostly well known for being an antagonist for ionotropic glutamate receptors and the neuroprotective effect this offers.

The KYN can also be converted to a second intermediate, 3-hydroxykynurenine, by the enzyme kynurenine-3-monooxygenase (KMO). Unlike the KYNA production step, this process is neurotoxic and decreases the concentration of KYNA [13]. The compound is further broken down into xanthurenic acid by KAT, which exhibits a weak glutamate agonist effect, or into a third intermediate compound, 3-hydroxyanthranilic acid, by the enzyme L-kynurenine hydrolase (KYNU). 3-hydroxyanthanilic acid is further broken down into picolinic acid, as well as quinolinic acid (QUIN), a neurotoxin but also the precursor to nicotinamide adenine dinucleotide (NAD^+^), an important coenzyme to mitochondrial metabolism of adenosine triphosphate (ATP).

In the nervous system, this KYN pathway swing between neuroprotection and neurotoxicity is of great interest. Physiological Trp metabolites (picolinic acid, kynurenic acid) have a neuroprotective effect, while high concentrations at pathophysiological levels contribute to neurotoxicity (quinolinic acid, 3-hydroxykinurenine) and compromised immuno-protective T-cell function [14]. This duality makes the KYN pathway a prime target for therapeutic development targeting neurological pathologies, including neuro-oncology, epilepsy, neurodegenerative diseases, and autoimmune disorders. Furthermore, emerging efforts are focused on developing KYN pathway-specific radiotracers. Under normal pathological conditions, Trp-based PET tracers show minimal brain accumulation, but radio-tagged kynurenine metabolites accumulate following brain injury or immune activation, suggesting the importance of the KYN pathway in pathological conditions [15].

Many CNS disorders have been shown to upregulate the enzyme IDO [16], which is a driver for increased Trp uptake. It is widely reported that the depletion of Trp and the resulting increase of its neurotoxic downstream metabolites caused by the upregulation of IDO in pathological conditions is toxic to T-cell functions and contributes to tumor immune resistance through an immunosuppressive effect on the T cells [17]. This points to the KYN pathway as a potential regulatory axis for the immune system, both during physiological and pathological conditions, including infection, autoimmunity, neuropathology, and oncology. Interest in the KYN pathway as a mechanism of the immune system has led to the development of many different PET radiopharmaceuticals targeting the KYN system to image these pathologies. Here we focus on reviewing KYN pathway-based radiotracers for brain disorders and oncology.

## 4. Tracer Types

**Figure 3:**
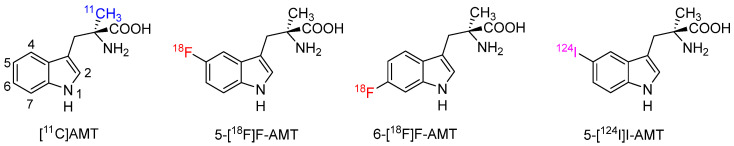
Chemical structures of [^11^C]AMT, 5-[^18^F]F-AMT, 6-[^18^F]F-AMT, and 5-[^124^I]I-AMT. The numbers in [^11^C]AMT denote possible radiolabeling sites on the indole ring.

Researchers have put tremendous efforts into developing pre-clinical Trp-based radiotracers with the goal of eventual clinical translation. Most Trp radiotracers are labeled by fluorine-18 or carbon-11 using the following typical strategies: (1) aliphatic nucleophilic substitution; (2) [^18^F]fluorination on the aromatic ring; (3) [^11^C]carbonyl reaction by [^11^C]carbon monoxide. Furthermore, radiotracers targeting immunosuppressive KYN pathway catalyzed by IDO or TDO under pathological conditions attract the most attention and are the focus of the current review. Early studies in *Escherichia coli* cultures with human IDO and TDO showed that the modification at the 5-position of Trp had minimal disturbance of the catalytic activity of IDO and TDO [18]. Therefore, many tracer designs focus on the 5-position to enhance the specificity of the KYN pathway. The naming conventions of these compounds relate to the radiopharmaceutical labeling strategies, with numbers referring to the position on the indole ring where the isotope is attached, with positions 1 through 3 on the pyrrole ring and positions 4 through 7 on the benzene ring as shown in Figure 3 [19].

### 4.1. α-11C-Methyl-L-Tryptophan ([^11^C]AMT), 5-^18^F-α-Methyl-L-Tryptophan (5-[^18^F]F-AMT), 6-^18^F-α-Methyl-L-Tryptophan (6-[^18^F]F-AMT), and 5-[^124^I]I-α-Methyl-Tryptophan (5-[^124^I]I-AMT)

The tracer [^11^C]AMT is one of the original radiotracers used as a Trp-based imaging agent primarily associated with the serotonergic pathway [20,21,22] (Figure 3), although it can be catabolized under pathological conditions in some cases by the KYN pathway as well [23]. Unlike many other Trp tracers, it has been studied extensively in both a clinical and pre-clinical setting. The tracer has been used to image numerous clinical pathologies such as depression, which are linked to the serotonergic system [24,25], and is also used to measure serotonin synthesis rates, both pre-clinically and clinically [26,27,28,29]. Serotonin synthesis levels have also been studied in patients with migraines, alcoholism, and suicidal tendencies using [^11^C]AMT [27,30,31]. It has also been used clinically to successfully differentiate between epileptogenic and non-epileptogenic tubers in patients with tuberous sclerosis complex and localize epileptic areas of the brain [32,33,34,35,36,37,38,39,40,41]. Localization of these epileptogenic foci has been linked to both increased serotonin synthesis and KYN pathway metabolism [42]. Many types of tumors including glioma, meningioma, non-small cell lung tumors, and breast cancer [23,43,44,45,46,47,48,49,50,51] have been imaged to great success in clinical trials, demonstrating the clinical potential of Trp-based tracers to image various tumor types. However, the carbon-11 with which this tracer is tagged, combined with the difficulty of synthesis, limits widespread clinical use because of its short half-life (20 min) and the necessity of cyclotron production. These factors make the study of Trp-based tracers challenging in all but the largest of institutions, which has driven researchers to focus on producing ^18^F-based radiotracers with a longer half-life and more flexibility to modify to improve KYN pathway specificity. Progress has been made in synthesizing ^18^F-labeled [^11^C]AMT analogs. Giglio et al. [52] replaced the 5-position hydrogen of the indole with a fluorine atom by a bioisosteric rationale and designed a [^11^C]AMT analog, 5-^18^F-α-methyl-L-tryptophan (5-[^18^F]F-AMT). This tracer has a similar structure to [^11^C]AMT but is produced with the commercially available fluorine-18, making it more accessible to local users. 5-[^18^F]F-AMT was radiosynthesized by a two-step copper-mediated [^18^F]fluorination approach with 10.9%–14.9% decay-corrected yield and molar activities of approximately 41 GBq/µmol. Initial cell- and enzyme-based assay showed 5-[^18^F]F-AMT was a substrate for IDO. Further study showed 5-[^18^F]F-AMT had a high tumor uptake in a B16F10 animal model and was a worse substrate for TPH compared with Trp and AMT. To expand the Trp radiotracers, the team synthesized 5-[^18^F]F-1-methyl-L-tryptophan and racemic 4-, 5-, 6-, 7-[^18^F]F-Trp by copper-catalyzed fluorodeboronylation strategy in 4.2%–14.9% decay-corrected yield, but no biological study data were provided. Krasikova et al. [53] reported a pre-clinical evaluation of 6-^18^F-α-methyl-L-tryptophan (6-[^18^F]F-AMT) and performed a head-to-head comparison between 6-[^18^F]F-AMT and [^11^C]AMT in mice. The undecay-corrected yield of 6-[^18^F]F-AMT was 7.5% with a molar activity of 58 GBq/µmol. Similar maximal brain radioactivity, regional brain uptake, and kinetic profiles were observed between the two tracers. However, 6-[^18^F]F-AMT demonstrated higher brain tissue background uptake of 5%–15% compared with [^11^C]AMT. The tracer was also reported to be a poor substrate for IDO or TPH using in silico docking studies, which was in contrast to 5-[^18^F]F-AMT and demonstrated that brain tissue uptake was more likely related to LAT1 transport than metabolism via the serotonin or KYN pathways.

Giglio et al. [54] reported radiolabeling of 5-[^124^I]I-AMT by a copper mediate isotope exchange strategy without protecting amino acid precursor. 5-[^124^I]I-AMT was obtained in 95% isolated yield but with a low molar activity of 27 GBq/µmol. 5-[^124^I]I-AMT was stable up to 20 h. An in vitro enzyme-based assay and ex vivo biodistribution in IDO1-positive B16F10 xenografts demonstrated 5-[^124^I]I-AMT was a promising KYN pathway agent targeting IDO1. PET imaging in tumor-bearing C57BL6 mice showed the tumor uptake was 3.24 ± 1.20, 3.42 ± 0.97% ID/g at 0.5 h, 1.4 h post-injection, respectively, while the kidney uptake was 27.93 ± 5.89, 22.52 ± 4.83% ID/g, respectively. Furthermore, the muscle showed minimal tracer accumulation; no apparent thyroid uptake was observed in vivo, indicating the stability of 5-[^124^I]I-AMT toward de-iodination.

### 4.2. ^11^C-5-Hydroxy-Typtophan ([^11^C]HTP)

**Figure 4:**
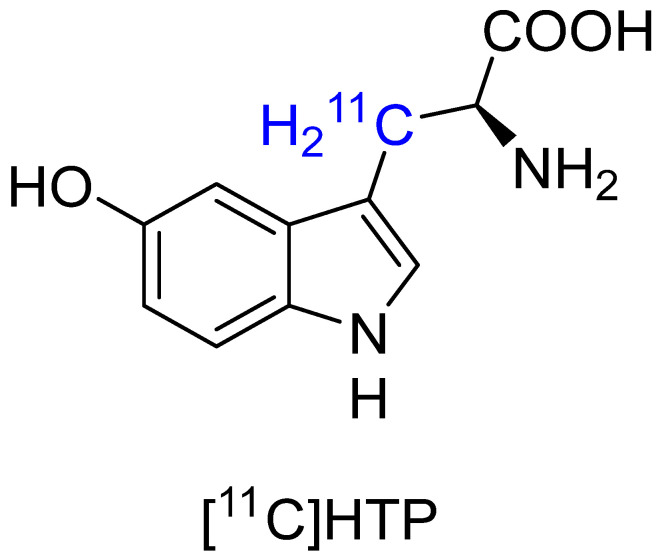
Chemical structure of [^11^C]HTP.

The tracer [^11^C]HTP has been used to successfully image neuroendocrine tumors using PET technology in clinical studies [55,56] (Figure 4). While it has more rapid metabolism than [^11^C]AMT because of more rapid cell membrane transport, its metabolism is more strongly associated with the serotonergic system [57], making it less than ideal for KYN pathway imaging. Unlike [^11^C]AMT, [^11^C]HTP does not significantly metabolize through the KYN pathway [58]. However, even in the case of activated KYN pathway, [^11^C]HTP can be metabolized by IDO but with a Michaelis-Menten constant (Km) 15 times higher than that of Trp, indicating hydroxylation at the 5-position of Trp decreases its affinity for the enzyme [59]. It is believed that [^11^C]HTP reflects serotonin-producing tumor status and can be used as a metabolic marker for neuroendocrine tumors, similar to FDG, given the tendency for neuroendocrine tumors to uptake and subsequently decarboxylate amine precursors via the serotonin pathway [60]. This is because, in many neuroendocrine tumors, the serotonin pathway is overactive [61]. Like [^11^C]AMT, [^11^C]HTP can be used for measuring serotonin synthesis [62]. However, [^11^C]HTP also had high non-specific accumulations in dopaminergic and noradrenergic presynaptic terminals because of its high affinity to AADC [57]. In addition, the short half-life of carbon-11 limits the use of this tracer to a small number of academic institutions.

In addition to [^11^C]AMT and its fluorine-18 analogs and [^11^C]HTP, most Trp radiotracers are fluorine-18 labeled and focus on modifications in three strategies: (1) fluoroalkoxy incorporation on the indole ring; (2) fluoroalkyl modification on the indole ring; (3) direct fluorination on the aromatic ring. The different side chain length and localization are expected to have a significant impact on the electronic properties, stability, metabolic pathways, in vivo pharmacokinetics, and tracer uptake mechanism (LAT, AADC, IDO/TDO mediated KYN pathway).

### 4.3. [^18^F]Fluoroalkoxy-Tryptophan

#### 4.3.1. L-5-(2-^18^F Fluorethoxy)-Tryptophan (5-^18^FEHTP)

**Figure 5:**
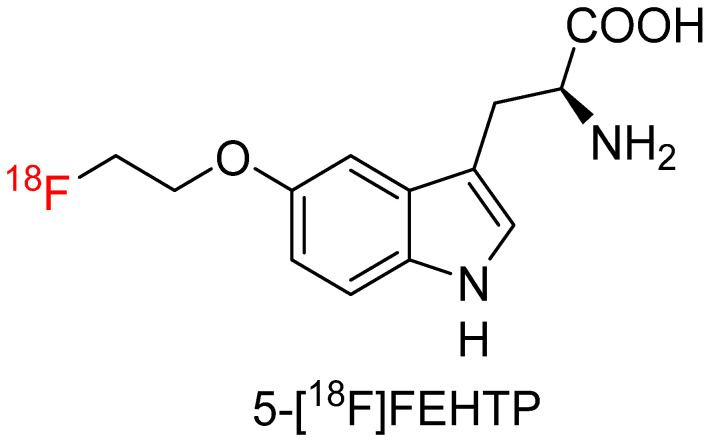
Chemical structure of 5-^18^FEHTP.

The labeling strategy for this compound uses a 2-[^18^F]fluoroethoxy group to attach the 5-position of the indole ring. Pre-clinical work has been done on the tracer ^18^F-FEHTrp by several research groups. It has been observed in S180 fibrosarcoma-inoculated and inflammation mice that high tumor uptake was observed for 5-^18^FEHTP and FDG at 1 h post-injection (Figure 5). However, the inflammatory side-to-background ratio was approximately 1.0 for 5-^18^FEHTP but over 3.0 for FDG, indicating 5-^18^FEHTP was superior to FDG in the differentiation of tumor from inflammation [63]. It has also been demonstrated to have a specificity as a substrate for the LAT1 transport mechanism but not a substrate of AADC in three pre-clinical xenograft-bearing mouse models of endocrine small cell lung cancer, pseudoendocrine prostate cancer, and exocrine breast cancer [64]. Abbas et al. [65] tried to use 5-^18^FEHTP to image LAT1 as a potential biomarker for accessing beta cell function in a mouse model of diabetes. Results showed 5-^18^FEHTP had the highest uptake in the pancreas of wild-type mice and significantly reduced uptake in endoplasmic reticulum stress-induced diabetic mice. However, in another group of mice with beta cell mass reduced by 62% with streptozotocin treatment, no 5-^18^FEHTP uptake difference was observed in the pancreas compared with the wild-type group. Therefore, 5-^18^FEHTP is not an appropriate radiotracer to noninvasively assess beta cell function during diabetes progression.

#### 4.3.2. D,L-4,6,7-(2-[^18^F]Fluorethoxy)-Tryptophan (4,6,7-^18^F-FEHTrp)

**Figure 6:**
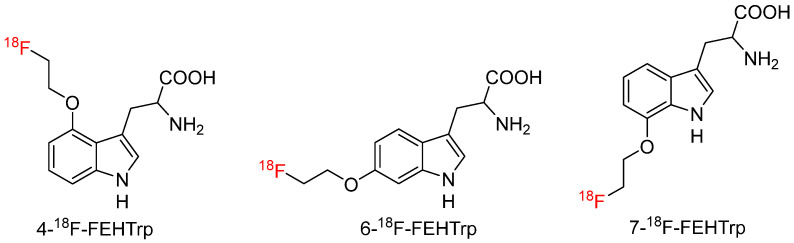
Chemical structures of racemic 4-^18^F-FEHTrp, 6-^18^F-FEHTrp, and 7-^18^F-FEHTrp.

In a pre-clinical study comparing racemic 4-, 6-, and 7-^18^F-FEHTrp [66] (Figure 6), 6-^18^F-FEHTrp was found to have the highest tumor-to-reference ratio (2.6 ± 0.2) via the LAT transport system in a small cell lung cancer xenograft model, but there was no evidence of tracer metabolism. Furthermore, 6-^18^F-FEHT outperformed the well-established tyrosine radiotracer, O-(2-[^18^F]fluoroethyl)-L-tyrosine (^18^F-FET).

#### 4.3.3. 5-(3-[^18^F]Fluoropropyloxy)-L-Tryptophan ([^18^F]-L-FPTP)

**Figure 7:**
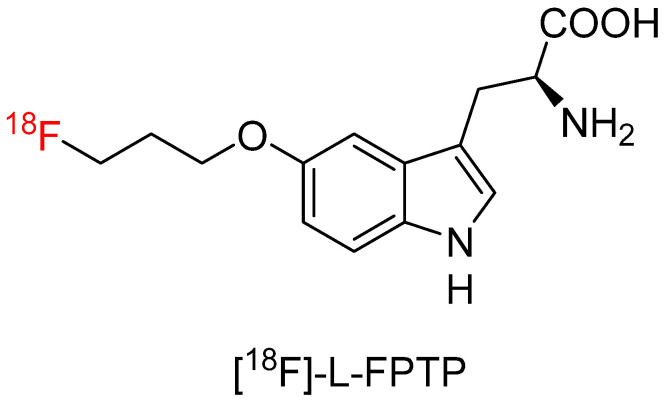
Chemical structure of [^18^F]-L-FPTP.

He et al. [67] developed an ^18^F-FEHTrp analog, 5-(3-[^18^F]Fluoropropyloxy)-L-tryptophan ([^18^F]-L-FPTP, Figure 7), with the 5-position of tryptophan modified by a 3-[^18^F]fluoropropyloxy group. [^18^F]-L-FPTP was radiosynthesized in 21.4% ± 4.4% undecay-corrected yield in 60 min. Cell uptake study showed [^18^F]-L-FPTP was not incorporated into protein and was transported by the amino acid transport system B^0,+^, LAT2, and ASC. PET imaging of [^18^F]-L-FPTP with tumor and inflammation xenografted in the same mice showed [^18^F]-L-FPTP had a high tumor-to-inflammation ratio (2.53) at 60 min post-injection. Furthermore, [^18^F]-L-FPTP had the highest tumor-to-inflammation ratio, but the lowest inflammation-to-muscle and inflammation-to-blood ratios compared with FDG and ^18^F-FEHTrp. Shih et al. [17] radiosynthesized racemic [^18^F]-L-FPTP in an automated module and evaluated the radiotracer in prostate cancer and small cell lung cancer animals. Results showed the tumor uptake was inferior to FDG in the prostate cancer model but higher than FDG in the small cell lung tumor model. However, no sample size and statistical analysis were provided for detailed comparison.

### 4.4. [^18^F]Fluoroalkyl-Tryptophan

#### 4.4.1. 1-(2-[^18^F]Fluoroethyl)-L-Tryptophan (^18^F-FETrp)

**Figure 8:**
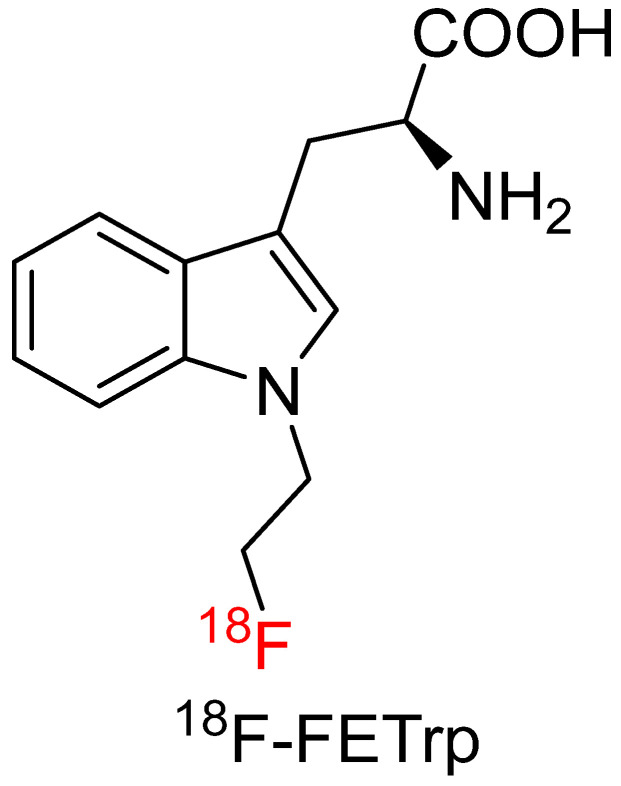
Chemical structure of ^18^F-FETrp.

^18^F-FETrp uses an [^18^F]fluoroethyl group to attach to 1-position of the indole ring (Figure 8). ^18^F-FETrp has gained plenty of interest as a potential radiotracer for the KYN pathway. ^18^F-FETrp has been demonstrated to have better tumor uptake than [^11^C]AMT in patient-derived xenograft (PDX) mouse models of glioblastoma, breast metastatic tumors, and non–small cell lung cancer metastatic tumors [68]. The tracer also shows promise in pre-clinical studies as an agent for imaging medulloblastoma with a tumor-to-reference ratio peaked at approximately 40 min post-injection of ^18^F-FETrp. ^18^F-FETrp also showed six times higher tumor uptake than its D-isomer at 60 min post-injection [69]. John et al. [70] reviewed 15 fluorine-18-labeled Trp radiotracers in radiochemistry, transport system, stability, biodistribution, tumor uptake, and metabolism pathways. The team concluded that [^18^F]FETrp and 5-[^18^F]F-AMT were the most promising candidates for clinical investigation. Both show more substrate activity with IDO compared with [^11^C]AMT, while potential in vivo defluorination exists for 5-[^18^F]F-AMT beyond 30 min post-injection. Obtaining highly enantiomeric pure L-isomer is essential for translational applications. Progress has been made in the synthesis of ^18^F-FETrp to where it can be produced in a commercial radiochemistry module with high radiochemical purity and enantiomeric values, paving the way for clinical studies at institutions without cyclotron access [6,71,72,73,74].

Recently, Muzik et al. [75] reported the first-in-human investigation of ^18^F-FETrp in two patients with gliomas and four with neuroendocrine tumors. A significantly different tumor uptake pattern was observed. ^18^F-FETrp peaked at 10 min post-injection in neuroendocrine tumors, followed by rapid washout, while the tracer accumulation in gliomas plateaued at around 40 min and showed heterogeneous uptake in the tumor regions. No adverse or clinically detectable pharmacologic effects were observed in all subjects. Biodistribution study from PET/CT scans showed urinary bladder had the highest tracer uptake followed by liver and kidneys. No significant in vivo defluorination was observed. Dosimetry study showed the urinary bladder received the highest radiation dose in male and female subjects. The clinical results demonstrated that ^18^F-FETrp had great promise to study the KYN pathway in vivo, particularly in solid tumors with IDO overexpression.

#### 4.4.2. 2-(3-[^18^F]Fluoropropyl)-DL-Tryptophan ([^18^F]2-FPTRP) and 5-(3-[^18^F]Fluoropropyl)-DL-Tryptophan ([^18^F]5-FPTRP)

**Figure 9:**
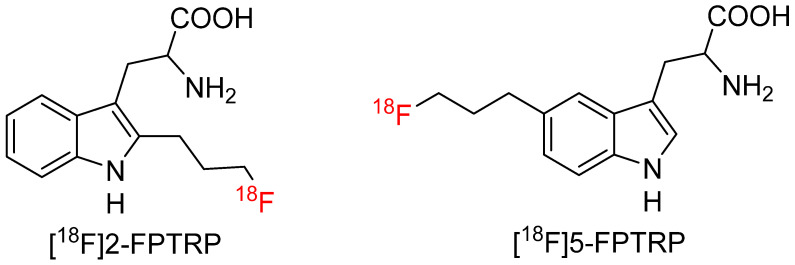
Chemical structures of racemic [^18^F]2-FPTRP and [^18^F]5-FPTRP.

Chiotellis et al. [76] reported two racemic-substituted Trp analogs with [^18^F]fluoropropyl substituted at 2-position ([^18^F]2-FPTRP) and 5-position ([^18^F]5-FPTRP), respectively (Figure 9). The two radiotracers were synthesized in 29%–34% decay-corrected yields and over 99% radiochemical purity. Both radiotracers have a molar activity of 30–82 GBq/µmol at the end of synthesis. In vitro cell uptake study at 37 °C showed 49% and 40% of the added [^18^F]2-FPTRP and [^18^F]5-FPTRP was accumulated within the small cell lung cancer cells in 5 min, respectively. The uptake was increased to 75% and 70% within 1 h, respectively. [^18^F]2-FPTRP and [^18^F]5-FPTRP showed higher cellular uptake than ^18^F-FEHTrp, indicating a fluoroethoxy substitution with a fluoropropyl group could enhance in vivo tumor uptake. The LAT inhibitor reduced the uptake of both radiotracers by 97% at 37 °C, suggesting that LAT is the predominant uptake mechanism for [^18^F]2-FPTRP and [^18^F]5-FPTRP in small cell lung cancer cells. PET imaging with [^18^F]2-FPTRP in two xenografts reached an SUV ratio of 2.4 at 60 min post-injection, which was remarkably higher than ^18^F-FEHTrp (SUV ratios < 2 in both models) and [^18^F]5-FPTRP (SUV ratio 1.8). The imaging findings were consistent with cellular uptake patterns. [^18^F]2-FPTRP also showed comparable tumor uptake to the well-established [^18^F]FET radiotracer but had a higher tumor-to-background ratio than [^18^F]5-FPTRP. Furthermore, the decarboxylase inhibitor S-carbidopa did not impact [^18^F]2-FPTRP uptake in small cell lung cancer xenograft, indicating [^18^F]2-FPTRP was not subjected to decarboxylation in vivo. Radiometabolite analysis confirmed a biotransformation step was not involved in tumor accumulation, supporting KYN pathway was not involved, and [^18^F]2-FPTRP was a promising PET radiotracer to study LAT activity in lesions.

#### 4.4.3. 5-Hydroxy-2-(3-[^18^F]Fluoropropyl)-DL-Tryptophan (5-OH-2-[^18^F]FPTRP) and 5-Hydroxy-2-(2-[^18^F]Fluoroethyl)-DL-Tryptophan (5-OH-2-[^18^F]FETRP)

**Figure 10:**
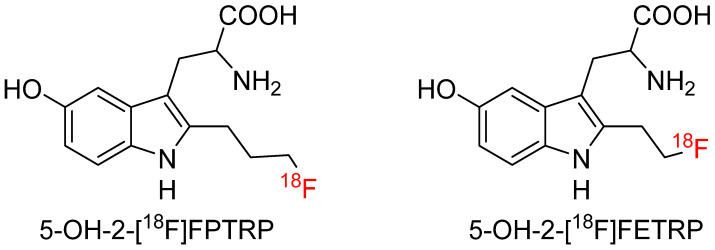
Chemical structures of racemic 5-OH-2-[^18^F]FPTRP and 5-OH-2-[^18^F]FETRP.

Chiotellis et al. [77] developed a new series of racemic Trp radiotracers by incorporating a free hydroxyl group into the 5-position of the indole ring but a [^18^F]fluoropropyl and a [^18^F]fluoroethyl in the 2-position (Figure 10). The team hypothesized that the 5-position hydroxyl group likely led to radiotracers more specific to AADC, similar to the well-known AADC substrate [^11^C]HTP. 5-OH-2-[^18^F]FPTRP and 5-OH-2-[^18^F]FETRP were radiosynthesized in 15%–39% radiochemical yield with molar activities of 45 to 95 GBq/µmol. In vitro cell uptake and enzyme studies showed the two tracers were taken up by LAT instead of AADC or IDO. Ex vivo metabolite study further confirmed only intact components were detected in the extracts from plasma, urine, and tumor samples at 60 min post-injection of both probes. The team evaluated the two probes in three animal models xenografted with human small cell lung cancer, human prostate cancer, and rat glioblastoma cell lines and compared the imaging results with [^18^F]FET. All three radiotracers reached the highest tumor-to-reference ratios between 60–75 min post-injection. However, both 5-OH-2-[^18^F]FPTRP and 5-OH-2-[^18^F]FETRP showed significantly lower muscle uptake but higher tumor-to-background ratio than [^18^F]FET in prostate cancer xenografts (ratios 4.3 ± 0.9, 3.9 ± 0.2, 1.8 ± 0.1, respectively). The two tracers also showed significantly higher bone-to-muscle ratios compared with [^18^F]FET (1.4 ± 0.13, 2.0 ± 0.5, 1.0 ± 0.1, respectively), indicating minor defluorination for 5-OH-2-[^18^F]FPTRP and 5-OH-2-[^18^F]FETRP.

### 4.5. [^18^F]Flourotryptophan

**Figure 11:**
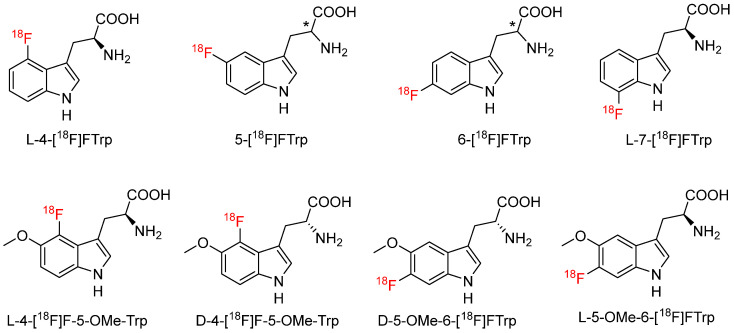
Chemical structures of [^18^F]fluorotryptophan with a fluorine-18 directly attached to the indole rings. * Designate as racemic or optically pure amino acids.

^18^F-FTrp has the fluorine bound directly to the indole ring (Figure 11). Early in the 1970s, Atkins et al. [78] first attempted to introduce fluorine-18 to tryptophan using the Balz–Schiemann reaction followed by hydrolysis. The team used racemic 5-[^18^F]F-Trp and 6-[^18^F]F-Trp to study their in vivo concentration and distribution in mice, rats, and dogs. Results showed the concentration of 6-[^18^F]F-Trp in the mouse pancreas was a function of the loading dose at 30 min post-injection (pancreas-to-liver ratio 12 at 16.1 mg/kg dose), but the concentration in the liver remained relatively stable. 5-[^18^F]F-Trp had less pancreas uptake than its 6-position radiotracer. Rats also showed high 6-[^18^F]F-Trp in the pancreas (pancreas-to-liver ratio 9 at 0.5 mg/kg dose). At 30 min post-injection of 6-[^18^F]F-Trp, about 37% of the radioactivity was excreted in mice while only 5% in rats. In dogs, 6-[^18^F]F-Trp uptake in the pancreas was low, and the pancreas-to-liver ratio was about 2 with a similar dose to mice and rats. Later on, Tang et al. [79] reported the radiosynthesis of L-5-[^18^F]fluorotryptophan (L-[^18^F]F-5-Trp) and D-5-[^18^F]fluorotryptophan (D-[^18^F]F-5-Trp) by a copper-mediated fluorodeboronylation strategy. Both tracers were produced in 1.5% ± 0.6% decay-corrected yield in approximately 200 min. The molar activity was 407–740 GBq/µmol and enantiomeric excess values of 90%–99%. The enzymatic assay showed L-[^18^F]F-5-Trp was an IDO1 and TDO2 substrate, whereas its D-counterpart was not. In vitro cell uptake studies showed L-[^18^F]F-5-Trp were human-IDO1 and TDO2 substrate with low Trp levels in media. However, in vivo PET imaging studies in mice bearing xenografts with various IDO1 expression levels showed the observed tumor uptake was not associated with tumor IDO1 activity. Furthermore, metabolite analysis showed the L-isomer was subject to extensive in vivo defluorination, making neither tracer appropriate to assess IDO/TDO activity. The team suggested 6-fluorotryptophan might have a different metabolite profile since it is a better substrate for TDO2 than Trp [80].

Schäfer et al. [81] radiosynthesized L-6-[^18^F]fluorotryptophan (L-[^18^F]F-6-Trp) by a two-step copper-mediated radiofluorination. L-[^18^F]F-6-Trp was produced in 16% ± 4% yield within 110 min with a molar activity of 280 GBq/µmol and enantiomeric excess of 89%. Weiss et al. [16] reported an ^18^F-^19^F isotopic exchange strategy to synthesize optically pure 4-[^18^F]F-L-Trp from a carbonyl-activated precursor containing a chiral moiety by three steps. 4-[^18^F]F-L-Trp was obtained in 13% radiochemical yield within 100 min. The enantiomeric excess was over 99% and molar activity of approximately 70 MBq/mmol. The low molar activity is typical for radiolabeling with an ^18^F-^19^F isotopic exchange strategy. No biological study was investigated in the reported study. During the development of [^11^C]AMT analog, 5-[^18^F]F-AMT, the researchers developed racemic 4-, 5-, 6-, 7-[^18^F]F-Trp but without biological evaluation [52]. Zlatopolskiy et al. [82] subsequently reported the preparation of optically pure 4-, 5-, 6-, 7-[^18^F]F-Trp by an alcohol-enhanced copper-mediated radiofluorination in 30%–53% yield. All tracers had high cellular uptake in glioblastoma, medulloblastoma, and estrogen receptors positive and estrogen receptors negative prostate cell lines. 4-, 5-, 6-[^18^F]F-Trps were stable in vitro but suffered from rapid in vivo defluorination, which may be explained by substrate specificity of IDO and TDO: all are substrates for IDO and TDO except 4-[^18^F]F-Trp (not a substrate of TDO). In contrast, 7-[^18^F]F-Trp displayed a high in vitro and in vivo stability. It accumulated in serotonergic areas of rat brains and melatonin-producing pineal gland. 7-[^18^F]F-Trp was also successfully used to image tumor xenografts in a chorioallantoic membrane xenograft model. 7-[^18^F]F-Trp is not an IDO or TDO substrate but an inhibitor of both enzymes. However, 7-^18^F-FTrp has been used to study Parkinson’s disease in a pre-clinical setting to investigate pathological alterations in the serotonergic, melatonin, and KYN pathways-associated with neuroinflammation [83]. The researchers stated a radiotracer specific to one pathway would benefit clinical application since different metabolism pathways may occur in the same brain areas.

More recently, Wu et al. [84] developed four enantiomeric pure [^11^C]AMT analogs, 4-F-5-OMe-tryptophans (L-4-[^18^F]F-5-OMe-Trp and D-4-[^18^F]F-5-OMe-Trp) and 5-OMe-6-F-tryptophans (L-5-OMe-6-[^18^F]F-Trp and D-5-OMe-6-[^18^F]F-Trp), through a novel photoredox radiofluorination strategy. L-4-[^18^F]F-5-OMe-Trp and its D-counterpart were radiosynthesized in 32.4% ± 4.1% and 26.1% ± 5.1% decay-corrected yields, respectively. However, the corresponding 6-position fluorine-18 isomers were obtained in 10 times lower yields than the 4-position analogs, probably due to reduced reactivity at 6-positions or competitive [^18^F]fluorination at 2- or 3-position. In vitro assays showed that L-4-[^18^F]F-5-OMe-Trp and L-5-OMe-6-[^18^F]F-Trp were substrates for the IDO1 enzyme, but none was a substrate for TPH. Both L-4-[^18^F]F-5-OMe-Trp and D-4-[^18^F]F-5-OMe-Trp showed substantial tumor uptake at 30 min post-injection in B16F10 tumor-bearing animal models and the L-isomer had much higher tumor uptake than its D-counterpart (9.58 ± 0.26% vs. 2.17 ± 0.10% ID/g). However, the 6-position L-isomer showed significant defluorination with low tumor-to-background contrast. In addition, the direct ^18^F-^19^F exchange had relatively lower molar activity, which may be addressed by replacing ^19^F in the radiolabeling substrates with other functional groups, such as chlorine. L-4-[^18^F]F-5-OMe-Trp showed good tumor-to-nontumor ratios and warranted further investigation. The representative KYN pathway-associated radiotracers are outlined in Table 1.

## 5. Discussion

There are many advantages to using Trp-based radiotracers to image brain pathology. Having high-quality localization of tumors or epileptogenic foci can be extremely valuable in surgical planning [85], which in turn will improve clinical outcomes for patients once the technology has matured. Perhaps the strongest advantage is that amino acid-based radiotracers can be used to image tumors that are hypometabolic and therefore have low glucose metabolism for FDG but do have uptake of Trp-based tracers because of the increased levels of IDO or TDO expressed by said tumors [86].

The importance of IDO expression in tumors cannot be understated, as this overexpression allows tumor cells to escape immune system surveillance by inhibiting immune cells through a combination of kynurenine metabolite accumulation and localized depletion of Trp [87]. The involvement of IDO/TDO in tumor immune system resistance represents a potential clinical opportunity for Trp-based tracers to assess IDO/TDO-mediated immune response. The connection between tumor immune resistance and the kynurenine system has excellent potential as a future line of research, with the potential to answer questions about how cancers can be more effectively treated. In addition, the KYN pathway is increasingly recognized as a key player in developing autoimmune disorders and psychiatric diseases [88,89,90,91,92]. The imbalance in the KYN pathway produces metabolites that can affect immune cell function and potentially contribute to pathological conditions, including multiple sclerosis, rheumatoid arthritis, schizophrenia, depression, anxiety, and neurodegenerative disorders. The degree of immune activation and the relationship between the KYN pathway and disease status may be assessed by measuring the levels of KYN, its metabolites, and KYN to its metabolite ratio. However, KYN pathway-based PET imaging is the preferred modality for stratifying patients, monitoring disease progression, and evaluating treatment response.

Increased transport into the cell via the LAT1 and LAT2 transporters [93] allows imaging of tumors that do not uptake more glucose than the surrounding brain tissue. Of special interest are neuroendocrine tumors, which tend to uptake amino acid precursors, with the subsequent decarboxylation, replacement of a carbon-bound carboxyl group with a hydrogen atom, trapping the radiotracer within the cells [94]. It is important to note that not all neuroendocrine tumors have low glucose metabolism, and the more aggressive tumors may still be well-imaged on PET using FDG [95]. Even in tumors with high glucose metabolism, imaging with FDG can have poor specificity when compared with amino acid-based tracers [96]. Because Trp-based tracers have a higher specificity than FDG, they play an important role in differentiating malignant cancers from benign conditions or other types of cancer with a different metabolism profile [97]. Because other amino acid-based tracers such as tyrosine have demonstrated increased transport and cellular uptake via LAT system, more clinical work is needed to determine whether Trp-based tracers have any clinical edge for LAT system imaging and their imaging contributions between LAT and KYN pathway.

Trp-based tracers also have significant advantages when it comes to imaging epileptic pathologies. Traditional PET imaging with FDG can show a hypometabolic zone during interictal imaging [98], which can be more difficult to interpret than Trp imaging. While the effectiveness of this technique has been measured using [^11^C]AMT, [^11^C]AMT has high uptake in epileptic foci regardless of ictal or interictal status. There is clinical potential for even better prognostic value using fluorine-18-based tracers because of the improved imaging characteristics of a longer-lived radiotracer, which makes the timing of epileptic imaging slightly more flexible for clinicians. Furthermore, more KYN pathway-specific radiotracers are emerging compared with the early developed and widely used [^11^C]AMT.

There are still many challenges in studying the KYN pathway, and one of the major concerns is the lack of TDO-specific radiotracers and current inability to differentiate between different IDO1, IDO2, and TDO substrates. Pre-clinical work has been done to measure IDO selectivity, with ^18^F-FETrp, 5-[^18^F]F-AMT, and L-4-[^18^F]F-5-OMe-Trp demonstrating the possibility to be good substrates for IDO using in vitro enzyme assays [70], while methods like immunohistochemistry (IHC) and reverse transcription-polymerase chain reaction (RT-PCR) can measure protein and mRNA expression, respectively [99]. These methods are invasive and cannot be used in vivo. Additional work has been done using ultraviolet-visible (UV-vis) spectroscopy to measure the UV absorbance peak of the KYN metabolite formylkynurenine to quantify the activity of IDO in vitro [80]. Measurements of serum ratios between Trp and KYN determine systemic Trp metabolism but do not localize IDO activity [99]. Further complicating matters, the serotonergic system has been linked to Trp depletion as well as a role in growth, progression, and angiogenic properties in certain tumors [100]. Pre-clinical work is being done on IDO inhibitors such as 1-methyl-tryptophan [101], which may help illuminate the situation with further study. These factors make it challenging to distinguish exact biochemical mechanisms using imaging alone, as many complex biochemical interactions influence the metabolism of Trp. Tremendous efforts have been focused on developing specific tryptophan radiotracers with a fluorine-18 radioisotope. ^18^F-FETrp is the most widely reported and is the only ^18^F-based tryptophan imaging agent under clinical investigations. However, both LAT and ASC systems are involved in the tumor cell uptake of ^18^F-FETrp. Inconsistent uptake of ^18^F-FETrp in the kidney and liver has been reported in animals. Tedious radiolabeling procedures are involved in obtaining high optically pure ^18^F-FETrp. In addition, only six patients were included in the first-in-human study. Remarkably different kinetics are observed in two types of tumors. A large sample size and multi-center clinical trials for various indications associated with the KYN pathway will further validate the potential of ^18^F-FETrp. Therefore, improved radiolabeling methods, more validations in different animal models, and extensive clinical trials are required to identify the radiotracers favorable for imaging the KYN pathway.

Many have speculated IDO inhibitors can be a valuable tool in reducing tumoral immune resistance, but investigations thus far have shown only limited effectiveness. Notably, a 2018 study demonstrated the failure of a late-stage IDO inhibitor to treat patients with metastatic melanoma [102], suggesting the role of the kynurenine pathway in oncology may be more complex than currently understood.

There has been pre-clinical work to differentiate between tracers that are selective for the LAT1 transporter or the LAT2 transporter. A technique for assessing LAT1 versus LAT2 transport has been developed using serine, a LAT2 substrate, to differentiate between these transporter types [103]. ^18^F-FEHTrp has been shown to be transported via the LAT1 transporter, while [^18^F]-L-FPTP is mainly transported via LAT2 [67]. Since amino acids are transported through the blood-brain barrier via LAT transporters [104], any neuroimaging depends on chemical compatibility of the tracer with the LAT transporter to some extent. Additional studies need to be done to more completely characterize the path of various Trp tracers through the LAT system.

While synthetic techniques for Trp radiotracers continue to improve, the process remains complex. Current labeling strategies either produce racemic Trp analogs or tend to produce both L and D enantiomers, requiring purification using a chiral HPLC column, which increases the compounding time and lowers radiochemical yield [105]. Additionally, the complex production of Trp-associated tracers currently limits clinical research to facilities with radiochemists capable of in-house multistage compounding of complex radiopharmaceuticals. This is somewhat of a roadblock for clinical translation, as it limits the study of these tracers to a small number of facilities, albeit a larger number than can effectively produce carbon-11 radiopharmaceuticals.

Another difficulty in Trp imaging is the defluorination of the radiotracers, which causes a certain amount of free fluoride to become unbonded from the Trp carrier molecules. This in vivo stability varies from tracer type to tracer type. For example, it was demonstrated that 4-, 5-, 6-[^18^F]FTrps suffer from rapid defluorination when compared with 7-[^18^F]FTrp in vivo [106]. It is hypothesized that radiotracer degradation and defluorination may be caused by enzymes in vivo and are typically measured on PET scans by monitoring bone uptake of the free [^18^F]fluoride. The exact mechanisms of tracer degradation are not fully understood currently.

PET is widely considered the most sensitive (nM to pM) imaging technique for noninvasively and quantitatively evaluating pathological conditions in living subjects in real time. However, PET has a relatively low spatial resolution for the body’s anatomy compared with CT or MRI. Therefore, PET/CT or PET/MRI can provide more accurate information than either scan alone. A PET scan is usually used alongside a CT or MRI scan. PET/CT and PET/MRI have complementary benefits for both pre-clinical research and clinical applications. Compared to PET/CT, PET/MR has a lower total ionizing radiation, which is a clear advantage in applications for pediatric diseases. For body PET/CT scans, the CT part typically consists of 60–80% of the radiation dose, while the PET radiopharmaceutical accounts for the remaining radiation dose [107,108]. In addition to their common functional imaging of PET/CT or PET/MRI at molecular levels, CT or MRI provides anatomical information with high spatial resolution but with a different focus. MRI has superior organ and soft tissue contrast, while CT shows better bone and lung imaging within a much faster scan time and a better ability to differentiate bone structures than MRI. Furthermore, simultaneous dual imaging modalities offer better image alignment and direct correlation. The integrated imaging technique in one scanning session also reduces the number of appointments, improving patient comfort.

## 6. Conclusions

The immunosuppressive KYN pathway plays a critical role in human pathophysiology. Noninvasive molecular imaging of the KYN pathway could provide valuable information for assessing cancer immunotherapy response, guiding epilepsy surgery, and stratifying patients. Additional work is needed to refine kynurenine system imaging. Future opportunities include performing more detailed biological studies to correlate gene expression with PET imaging findings, validating kinetic modeling to monitor treatment responses, and developing tracers with a higher specificity for IDO and TDO. While extensive pre-clinical work has been reported on the development of ^18^F-based Trp tracers, there has yet to be a demonstrated consensus on which tracer is the best for clinical translation. More extensive clinical investigations with ^18^F-FETrp to replicate the clinical findings reported with [^11^C]AMT are urgently needed, while a first-in-human investigation with ^18^F-FETrp shows promising imaging and safety results. Fluorine-18 has a half-life of 110 min, which is a substantial improvement over the 20-min half-life of carbon-11; however, the complex production and purification of Trp-based tracers still takes time and makes ready automation and compounding a legitimate burden to the clinical translation of the research, despite the improved working duration of fluorine 18-based tracers over their carbon-11 predecessors. Developing tracers that are highly specific to the kynurenine pathway with low defluorination and manageable synthesis has been a high priority [109], but pre-clinical work is continuing in the hope of exploring the ideal Trp tracers for PET imaging. Despite this, many potential clinical applications of Trp-based imaging agents are moving forward, especially in the clinical areas of brain tumors, epilepsy, and neuroinflammation. As the synthesis and availability of KYN pathway tracers improve, there will undoubtedly be more widespread clinical translation.

## Figures and Tables

**Table 1:** A summary of representative KYN pathway radiotracers.

Tracer Name	Pre-Clinical/Clinical	Application	IDO/TDO Substrate	Pros	Cons
[^11^C]AMT	Pre-clinical and clinical	Serotonin synthesis measurement Oncology Epilepsy Neuropsychiatric disorders	IDO/TDO	IDO/TDO substrate Not incorporated into protein Better imaging than FDG	Short half-life Challenging for radiolabeling Non-specific serotonin pathway Available from limited centers
[^11^C]HTP	Pre-clinical and clinical	Neuroendocrine tumors Serotonin synthesis measurement	Limited IDO	Widely used in clinical investigations	Short half-life Mainly metabolized through the serotonergic system Non-specific substrate for AADC
5-[^18^F]F-AMT	Pre-clinical	Oncology	IDO	Favorable half-life A poor substrate for TPH More IDO specific than [^11^C]AMT	Potential defluorination at later imaging period
5-[^124^I]I-AMT	Pre-clinical	Oncology	IDO	Block protein synthesis pathway Long half-life Easy to switch between imaging and therapy	Low molar activity Clearance fast, need matched radionuclide half-life
^18^F-FETrp	Pre-clinical and clinical	Oncology	IDO	Favorable half-life First-in-human investigation Most promising IDO-targeting tracers More IDO specific than [^11^C]AMT	Complex procedures to obtain optically pure L-isomer in an automatic radiolabeling system
L-[^18^F]F-4-Trp	Pre-clinical	Oncology	IDO	Favorable half-life Stable in vitro	Rapid in vivo defluorination
L-[^18^F]F-5-Trp	Pre-clinical	Oncology	IDO/TDO	IDO/TDO substrate in vitro at low Trp concentration Stable in vitro	Not an IDO/TDO substrate in vivo Rapid in vivo defluorination
L-[^18^F]F-6-Trp	Pre-clinical	Oncology	IDO/TDO	Favorable half-life Stable in vitro	Rapid in vivo defluorination
L-[^18^F]F-7-Trp	Pre-clinical	Oncology Parkinson’s disease	IDO/TDO	Favorable half-life High in vitro and in vivo stability	Non-specific substrate for serotonergic, melatonin, and KYN pathways Exact pathway debatable
L-4-[^18^F]F-5-OMe-Trp	Pre-clinical	Oncology	IDO	Not a substrate for TPH	Low molar activity
L-6-[^18^F]F-5-OMe-Trp	Pre-clinical	Oncology	IDO	Not a substrate for TPH	Significant defluorination Low tumor-to-background contrast Low molar activity

## Data Availability

The materials and resources in this study are available from the corresponding author upon request.

## References

[1] Xue C., Li G., Zheng Q., Gu X., Shi Q., Su Y., Chu Q., Yuan X., Bao Z., Lu J. (2023). Tryptophan metabolism in health and disease. Cell Metab..

[2] Liu X.H., Zhai X.Y. (2021). Role of tryptophan metabolism in cancers and therapeutic implications. Biochimie.

[3] Shankar A., Bomanji J., Hyare H. (2020). Hybrid PET-MRI Imaging in Paediatric and TYA Brain Tumours: Clinical Applications and Challenges. J. Pers. Med..

[4] Juhasz C., Dwivedi S., Kamson D.O., Michelhaugh S.K., Mittal S. (2014). Comparison of amino acid positron emission tomographic radiotracers for molecular imaging of primary and metastatic brain tumors. Mol. Imaging.

[5] Sun T., Tang G., Tian H., Wang X., Chen X., Chen Z., Wang S. (2012). Radiosynthesis of 1-[18F]fluoroethyl-L-tryptophan as a novel potential amino acid PET tracer. Appl. Radiat. Isot..

[6] Jiang H., Guo Y., Cai H., Viola N., Shields A.F., Muzik O., Juhasz C. (2023). Automated radiosynthesis of 1-(2-[(18) F]fluoroethyl)-L-tryptophan ([(18) F]FETrp) for positron emission tomography (PET) imaging of cancer in humans. J. Labelled Comp. Radiopharm..

[7] Henrottin J., Lemaire C., Egrise D., Zervosen A., Van den Eynde B., Plenevaux A., Franci X., Goldman S., Luxen A. (2016). Fully automated radiosynthesis of N(1)-[(18)F]fluoroethyl-tryptophan and study of its biological activity as a new potential substrate for indoleamine 2,3-dioxygenase PET imaging. Nucl. Med. Biol..

[8] Koopmans K.P., Neels O.N., Kema I.P., Elsinga P.H., Links T.P., de Vries E.G., Jager P.L. (2009). Molecular imaging in neuroendocrine tumors: Molecular uptake mechanisms and clinical results. Crit. Rev. Oncol. Hematol..

[9] Saito Y., Soga T. (2021). Amino acid transporters as emerging therapeutic targets in cancer. Cancer Sci..

[10] Wang Q., Holst J. (2015). L-type amino acid transport and cancer: Targeting the mTORC1 pathway to inhibit neoplasia. Am. J. Cancer Res..

[11] Vumma R., Johansson J., Lewander T., Venizelos N. (2011). Tryptophan transport in human fibroblast cells-a functional characterization. Int. J. Tryptophan Res..

[12] Tronel C., Largeau B., Santiago Ribeiro M.J., Guilloteau D., Dupont A.C., Arlicot N. (2017). Molecular Targets for PET Imaging of Activated Microglia: The Current Situation and Future Expectations. Int. J. Mol. Sci..

[13] Chen Y., Zhang J., Yang Y., Xiang K., Li H., Sun D., Chen L. (2022). Kynurenine-3-monooxygenase (KMO): From its biological functions to therapeutic effect in diseases progression. J. Cell Physiol..

[14] Braidy N., Grant R., Brew B.J., Adams S., Jayasena T., Guillemin G.J. (2009). Effects of Kynurenine Pathway Metabolites on Intracellular NAD Synthesis and Cell Death in Human Primary Astrocytes and Neurons. Int. J. Tryptophan Res..

[15] Goud N.S., Bhattacharya A., Joshi R.K., Nagaraj C., Bharath R.D., Kumar P. (2021). Carbon-11: Radiochemistry and Target-Based PET Molecular Imaging Applications in Oncology, Cardiology, and Neurology. J. Med. Chem..

[16] Weiss P.S., Ermert J., Castillo Melean J., Schafer D., Coenen H.H. (2015). Radiosynthesis of 4-[(18)F]fluoro-L-tryptophan by isotopic exchange on carbonyl-activated precursors. Bioorg Med. Chem..

[17] Shih I.H., Duan X.D., Kong F.L., Williams M.D., Yang K., Zhang Y.H., Yang D.J. (2014). Automated synthesis of 18F-fluoropropoxytryptophan for amino acid transporter system imaging. Biomed. Res. Int..

[18] Basran J., Rafice S.A., Chauhan N., Efimov I., Cheesman M.R., Ghamsari L., Raven E.L. (2008). A kinetic, spectroscopic, and redox study of human tryptophan 2,3-dioxygenase. Biochemistry.

[19] Chehardoli G., Bahmani A. (2021). Synthetic strategies, SAR studies, and computer modeling of indole 2 and 3-carboxamides as the strong enzyme inhibitors: A review. Mol. Divers..

[20] Chugani D.C., Muzik O. (2000). Alpha[C-11]methyl-L-tryptophan PET maps brain serotonin synthesis and kynurenine pathway metabolism. J. Cereb. Blood Flow. Metab..

[21] Rosa-Neto P., Diksic M., Leyton M., Mzengeza S., Benkelfat C. (2005). Stability of alpha-[11C]methyl-L-tryptophan brain trapping in healthy male volunteers. Eur. J. Nucl. Med. Mol. Imaging.

[22] Rosa-Neto P., Benkelfat C., Sakai Y., Leyton M., Morais J.A., Diksic M. (2007). Brain regional alpha-[11C]methyl-L-tryptophan trapping, used as an index of 5-HT synthesis, in healthy adults: Absence of an age effect. Eur. J. Nucl. Med. Mol. Imaging.

[23] Juhasz C., Bosnyak E. (2016). PET and SPECT studies in children with hemispheric low-grade gliomas. Childs Nerv. Syst..

[24] Rosa-Neto P., Diksic M., Okazawa H., Leyton M., Ghadirian N., Mzengeza S., Nakai A., Debonnel G., Blier P., Benkelfat C. (2004). Measurement of brain regional alpha-[11C]methyl-L-tryptophan trapping as a measure of serotonin synthesis in medication-free patients with major depression. Arch. Gen. Psychiatry.

[25] Berney A., Nishikawa M., Benkelfat C., Debonnel G., Gobbi G., Diksic M. (2008). An index of 5-HT synthesis changes during early antidepressant treatment: Alpha-[11C]methyl-L-tryptophan PET study. Neurochem. Int..

[26] Okazawa H., Nishizawa S., Tsuchida T., Yonekura Y., Diksic M. (2004). A simplified autoradiographic method with alpha-[14C]methyl-tryptophan to measure serotonin synthesis rate in the rat brain. Nucl. Med. Biol..

[27] Sakai Y., Nishikawa M., Leyton M., Benkelfat C., Young S.N., Diksic M. (2006). Cortical trapping of alpha-[(11)C]methyl-l-tryptophan, an index of serotonin synthesis, is lower in females than males. Neuroimage.

[28] Kumakura Y., Natsume J., Toussaint P.J., Nakai A., Rosa-Neto P., Meyer E., Diksic M. (2011). Generation of Functional Images of The Brain Trapping Constant for alpha-[11C]Methyl-L-Tryptophan Using Constrained Linear Regression. Open J. Med. Imaging.

[29] Frey B.N., Skelin I., Sakai Y., Nishikawa M., Diksic M. (2010). Gender differences in alpha-[(11)C]MTrp brain trapping, an index of serotonin synthesis, in medication-free individuals with major depressive disorder: A positron emission tomography study. Psychiatry Res..

[30] Nishikawa M., Diksic M., Sakai Y., Kumano H., Charney D., Palacios-Boix J., Negrete J., Gill K. (2009). Alterations in brain serotonin synthesis in male alcoholics measured using positron emission tomography. Alcohol. Clin. Exp. Res..

[31] Leyton M., Paquette V., Gravel P., Rosa-Neto P., Weston F., Diksic M., Benkelfat C. (2006). alpha-[11C]Methyl-L-tryptophan trapping in the orbital and ventral medial prefrontal cortex of suicide attempters. Eur. Neuropsychopharmacol..

[32] Rintahaka P.J., Chugani H.T. (1997). Clinical role of positron emission tomography in children with tuberous sclerosis complex. J. Child. Neurol..

[33] Kumar A., Asano E., Chugani H.T. (2011). alpha-[(1)(1)C]-methyl-L-tryptophan PET for tracer localization of epileptogenic brain regions: Clinical studies. Biomark. Med..

[34] Luat A.F., Makki M., Chugani H.T. (2007). Neuroimaging in tuberous sclerosis complex. Curr. Opin. Neurol..

[35] Rubi S., Costes N., Heckemann R.A., Bouvard S., Hammers A., Marti Fuster B., Ostrowsky K., Montavont A., Jung J., Setoain X. (2013). Positron emission tomography with alpha-[11C]methyl-L-tryptophan in tuberous sclerosis complex-related epilepsy. Epilepsia.

[36] Chugani H.T., Juhasz C., Chugani D.C., Lawrenson L., Muzik O., Chakraborty P.K., Sood S. (2008). Increased striatal serotonin synthesis following cortical resection in children with intractable epilepsy. Epilepsy Res..

[37] Kumar A., Chugani H.T. (2017). The Role of Radionuclide Imaging in Epilepsy, Part 2: Epilepsy Syndromes. J. Nucl. Med. Technol..

[38] Juhasz C., Chugani H.T. (2003). Imaging the epileptic brain with positron emission tomography. Neuroimaging Clin. N. Am..

[39] Juhasz C., Chugani D.C., Muzik O., Shah A., Asano E., Mangner T.J., Chakraborty P.K., Sood S., Chugani H.T. (2003). Alpha-methyl-L-tryptophan PET detects epileptogenic cortex in children with intractable epilepsy. Neurology.

[40] Chugani H.T., Luat A.F., Kumar A., Govindan R., Pawlik K., Asano E. (2013). alpha-[11C]-Methyl-L-tryptophan--PET in 191 patients with tuberous sclerosis complex. Neurology.

[41] Wakamoto H., Chugani D.C., Juhasz C., Muzik O., Kupsky W.J., Chugani H.T. (2008). Alpha-methyl-l-tryptophan positron emission tomography in epilepsy with cortical developmental malformations. Pediatr. Neurol..

[42] Fedi M., Reutens D.C., Andermann F., Okazawa H., Boling W., White C., Dubeau F., Nakai A., Gross D.W., Andermann E. (2003). alpha-[11C]-Methyl-L-tryptophan PET identifies the epileptogenic tuber and correlates with interictal spike frequency. Epilepsy Res..

[43] Bosnyak E., Barger G.R., Michelhaugh S.K., Robinette N.L., Amit-Yousif A., Mittal S., Juhasz C. (2018). Amino Acid PET Imaging of the Early Metabolic Response During Tumor-Treating Fields (TTFields) Therapy in Recurrent Glioblastoma. Clin. Nucl. Med..

[44] Juhasz C., Chugani D.C., Muzik O., Wu D., Sloan A.E., Barger G., Watson C., Shah A.K., Sood S., Ergun E.L. (2006). In vivo uptake and metabolism of alpha-[11C]methyl-L-tryptophan in human brain tumors. J. Cereb. Blood Flow. Metab..

[45] Juhasz C., Muzik O., Chugani D.C., Chugani H.T., Sood S., Chakraborty P.K., Barger G.R., Mittal S. (2011). Differential kinetics of alpha-[(1)(1)C]methyl-L-tryptophan on PET in low-grade brain tumors. J. Neurooncol.

[46] Kamson D.O., Juhasz C., Buth A., Kupsky W.J., Barger G.R., Chakraborty P.K., Muzik O., Mittal S. (2013). Tryptophan PET in pretreatment delineation of newly-diagnosed gliomas: MRI and histopathologic correlates. J. Neurooncol.

[47] Kamson D.O., Mittal S., Robinette N.L., Muzik O., Kupsky W.J., Barger G.R., Juhasz C. (2014). Increased tryptophan uptake on PET has strong independent prognostic value in patients with a previously treated high-grade glioma. Neuro Oncol..

[48] Juhasz C., Muzik O., Lu X., Jahania M.S., Soubani A.O., Khalaf M., Peng F., Mangner T.J., Chakraborty P.K., Chugani D.C. (2009). Quantification of tryptophan transport and metabolism in lung tumors using PET. J. Nucl. Med..

[49] Alkonyi B., Barger G.R., Mittal S., Muzik O., Chugani D.C., Bahl G., Robinette N.L., Kupsky W.J., Chakraborty P.K., Juhasz C. (2012). Accurate differentiation of recurrent gliomas from radiation injury by kinetic analysis of alpha-11C-methyl-L-tryptophan PET. J. Nucl. Med..

[50] Peng F., Juhasz C., Bhambhani K., Wu D., Chugani D.C., Chugani H.T. (2007). Assessment of progression and treatment response of optic pathway glioma with positron emission tomography using alpha-[(11)C]methyl-L-tryptophan. Mol. Imaging Biol..

[51] Juhasz C., Nahleh Z., Zitron I., Chugani D.C., Janabi M.Z., Bandyopadhyay S., Ali-Fehmi R., Mangner T.J., Chakraborty P.K., Mittal S. (2012). Tryptophan metabolism in breast cancers: Molecular imaging and immunohistochemistry studies. Nucl. Med. Biol..

[52] Giglio B.C., Fei H., Wang M., Wang H., He L., Feng H., Wu Z., Lu H., Li Z. (2017). Synthesis of 5-[(18)F]Fluoro-alpha-methyl Tryptophan: New Trp Based PET Agents. Theranostics.

[53] Krasikova R., Kondrashov M., Avagliano C., Petukhov M., Vazquez-Romero A., Revunov E., Johnstrom P., Tari L., Toth M., Haggkvist J. (2020). Synthesis and Preclinical Evaluation of 6-[(18)F]Fluorine-alpha-methyl-l-tryptophan, a Novel PET Tracer for Measuring Tryptophan Uptake. ACS Chem. Neurosci..

[54] Giglio B.C., Wang H., Yan X., Li Z. (2019). Synthesis and initial evaluation of radioactive 5-I-alpha-methyl-tryptophan: A Trp based agent targeting IDO-1. Medchemcomm.

[55] Orlefors H., Sundin A., Garske U., Juhlin C., Oberg K., Skogseid B., Langstrom B., Bergstrom M., Eriksson B. (2005). Whole-body (11)C-5-hydroxytryptophan positron emission tomography as a universal imaging technique for neuroendocrine tumors: Comparison with somatostatin receptor scintigraphy and computed tomography. J. Clin. Endocrinol. Metab..

[56] Nikolaou A., Thomas D., Kampanellou C., Alexandraki K., Andersson L.G., Sundin A., Kaltsas G. (2010). The value of 11C-5-hydroxy-tryptophan positron emission tomography in neuroendocrine tumor diagnosis and management: Experience from one center. J. Endocrinol. Investig..

[57] Lundquist P., Hartvig P., Blomquist G., Hammarlund-Udenaes M., Langstrom B. (2007). 5-Hydroxy-L-[beta-11C]tryptophan versus alpha-[11C]methyl-L-tryptophan for positron emission tomography imaging of serotonin synthesis capacity in the rhesus monkey brain. J. Cereb. Blood Flow. Metab..

[58] Visser A.K., van Waarde A., Willemsen A.T., Bosker F.J., Luiten P.G., den Boer J.A., Kema I.P., Dierckx R.A. (2011). Measuring serotonin synthesis: From conventional methods to PET tracers and their (pre)clinical implications. Eur. J. Nucl. Med. Mol. Imaging.

[59] Yamazaki F., Kuroiwa T., Takikawa O., Kido R. (1985). Human indolylamine 2,3-dioxygenase. Its tissue distribution, and characterization of the placental enzyme. Biochem. J..

[60] van Essen M., Sundin A., Krenning E.P., Kwekkeboom D.J. (2014). Neuroendocrine tumours: The role of imaging for diagnosis and therapy. Nat. Rev. Endocrinol..

[61] Koopmans K.P., Glaudemans A.W. (2014). Other PET tracers for neuroendocrine tumors. PET Clin..

[62] Lopresti B.J., Royse S.K., Mathis C.A., Tollefson S.A., Narendran R. (2023). Beyond monoamines: I. Novel targets and radiotracers for Positron emission tomography imaging in psychiatric disorders. J. Neurochem..

[63] Li R., Wu S.C., Wang S.C., Fu Z., Dang Y., Huo L. (2010). Synthesis and evaluation of l-5-(2-[(18)F]fluoroethoxy)tryptophan as a new PET tracer. Appl. Radiat. Isot..

[64] Kramer S.D., Mu L., Muller A., Keller C., Kuznetsova O.F., Schweinsberg C., Franck D., Muller C., Ross T.L., Schibli R. (2012). 5-(2-18F-fluoroethoxy)-L-tryptophan as a substrate of system L transport for tumor imaging by PET. J. Nucl. Med..

[65] Abbas A., Beamish C., McGirr R., Demarco J., Cockburn N., Krokowski D., Lee T.Y., Kovacs M., Hatzoglou M., Dhanvantari S. (2016). Characterization of 5-(2-(18)F-fluoroethoxy)-L-tryptophan for PET imaging of the pancreas. F1000Res.

[66] Chiotellis A., Muller A., Mu L., Keller C., Schibli R., Kramer S.D., Ametamey S.M. (2014). Synthesis and biological evaluation of (18)F-labeled Fluoroethoxy tryptophan analogues as potential PET tumor imaging agents. Mol. Pharm..

[67] He S., Tang G., Hu K., Wang H., Wang S., Huang T., Liang X., Tang X. (2013). Radiosynthesis and biological evaluation of 5-(3-[18F]fluoropropyloxy)-L-tryptophan for tumor PET imaging. Nucl. Med. Biol..

[68] Michelhaugh S.K., Muzik O., Guastella A.R., Klinger N.V., Polin L.A., Cai H., Xin Y., Mangner T.J., Zhang S., Juhasz C. (2017). Assessment of Tryptophan Uptake and Kinetics Using 1-(2-18F-Fluoroethyl)-l-Tryptophan and alpha-11C-Methyl-l-Tryptophan PET Imaging in Mice Implanted with Patient-Derived Brain Tumor Xenografts. J. Nucl. Med..

[69] Xin Y., Yue X., Li H., Li Z., Cai H., Choudhary A.K., Zhang S., Chugani D.C., Langhans S.A. (2020). PET imaging of medulloblastoma with an (18)F-labeled tryptophan analogue in a transgenic mouse model. Sci. Rep..

[70] John F., Muzik O., Mittal S., Juhasz C. (2020). Fluorine-18-Labeled PET Radiotracers for Imaging Tryptophan Uptake and Metabolism: A Systematic Review. Mol. Imaging Biol..

[71] Xin Y., Cai H. (2017). Improved Radiosynthesis and Biological Evaluations of L- and D-1-[(18)F]Fluoroethyl-Tryptophan for PET Imaging of IDO-Mediated Kynurenine Pathway of Tryptophan Metabolism. Mol. Imaging Biol..

[72] Xin Y., Gao X., Liu L., Ge W.P., Jain M.K., Cai H. (2019). Evaluation of L-1-[(18)F]Fluoroethyl-Tryptophan for PET Imaging of Cancer. Mol. Imaging Biol..

[73] Maisonial-Besset A., Kryza D., Kopka K., Levesque S., Moreau E., Wenzel B., Chezal J.M. (2024). Improved automated one-pot two-step radiosynthesis of (S)-[(18)F]FETrp, a radiotracer for PET imaging of indoleamine 2,3-dioxygenase 1 (IDO1). EJNMMI Radiopharm. Chem..

[74] Yue X., Nikam R.M., Kecskemethy H.H., Kandula V.V.R., Falchek S.J., Averill L.W., Langhans S.A. (2021). Radiosynthesis of 1-(2-[18F]Fluoroethyl)-L-Tryptophan using a One-pot, Two-step Protocol. J. Vis. Exp..

[75] Muzik O., Shields A.F., Barger G.R., Jiang H., Chamiraju P., Juhasz C. (2024). The First Human Application of an F-18-Labeled Tryptophan Analog for PET Imaging of Cancer. Mol. Imaging Biol..

[76] Chiotellis A., Mu L., Muller A., Selivanova S.V., Keller C., Schibli R., Kramer S.D., Ametamey S.M. (2013). Synthesis and biological evaluation of (1)(8)F-labeled fluoropropyl tryptophan analogs as potential PET probes for tumor imaging. Eur. J. Med. Chem..

[77] Chiotellis A., Muller Herde A., Rossler S.L., Brekalo A., Gedeonova E., Mu L., Keller C., Schibli R., Kramer S.D., Ametamey S.M. (2016). Synthesis, Radiolabeling, and Biological Evaluation of 5-Hydroxy-2-[(18)F]fluoroalkyl-tryptophan Analogues as Potential PET Radiotracers for Tumor Imaging. J. Med. Chem..

[78] Atkins H.L., Christman D.R., Fowler J.S., Hauser W., Hoyte R.M., Klopper J.F., Lin S.S., Wolf A.P. (1972). Organic radiopharmaceuticals labeled with isotopes of short half-life. V. 18 F-labeled 5- and 6-fluorotryptophan. J. Nucl. Med..

[79] Tang T., Gill H.S., Ogasawara A., Tinianow J.N., Vanderbilt A.N., Williams S.P., Hatzivassiliou G., White S., Sandoval W., DeMent K. (2017). Preparation and evaluation of L- and D-5-[(18)F]fluorotryptophan as PET imaging probes for indoleamine and tryptophan 2,3-dioxygenases. Nucl. Med. Biol..

[80] Leeds J.M., Brown P.J., McGeehan G.M., Brown F.K., Wiseman J.S. (1993). Isotope effects and alternative substrate reactivities for tryptophan 2,3-dioxygenase. J. Biol. Chem..

[81] Schäfer D., Weiß P., Ermert J., Castillo Meleán J., Zarrad F., Neumaier B. (2016). Preparation of No-Carrier-Added 6-[18F]Fluoro-l-tryptophan via Cu-Mediated Radiofluorination. Eur. J. Org. Chem..

[82] Zlatopolskiy B.D., Zischler J., Schafer D., Urusova E.A., Guliyev M., Bannykh O., Endepols H., Neumaier B. (2018). Discovery of 7-[(18)F]Fluorotryptophan as a Novel Positron Emission Tomography (PET) Probe for the Visualization of Tryptophan Metabolism in Vivo. J. Med. Chem..

[83] Endepols H., Zlatopolskiy B.D., Zischler J., Alavinejad N., Apetz N., Vus S., Drzezga A., Neumaier B. (2022). Imaging of cerebral tryptophan metabolism using 7-[(18)F]FTrp-PET in a unilateral Parkinsonian rat model. Neuroimage.

[84] Wu X., Ma X., Zhong Y., Chen W., Xu M., Zhang H., Wang L., Tu X., Han Z., Zhao W. (2023). Development of [(18)F]F-5-OMe-Tryptophans through Photoredox Radiofluorination: A New Method to Access Tryptophan-Based PET Agents. J. Med. Chem..

[85] Patil S., Biassoni L., Borgwardt L. (2007). Nuclear medicine in pediatric neurology and neurosurgery: Epilepsy and brain tumors. Semin. Nucl. Med..

[86] Alkonyi B., Mittal S., Zitron I., Chugani D.C., Kupsky W.J., Muzik O., Chugani H.T., Sood S., Juhasz C. (2012). Increased tryptophan transport in epileptogenic dysembryoplastic neuroepithelial tumors. J. Neurooncol.

[87] Uyttenhove C., Pilotte L., Theate I., Stroobant V., Colau D., Parmentier N., Boon T., Van den Eynde B.J. (2003). Evidence for a tumoral immune resistance mechanism based on tryptophan degradation by indoleamine 2,3-dioxygenase. Nat. Med..

[88] Kenney L.L., Chiu R.S., Dutra M.N., Wactor A., Honan C., Shelerud L., Corrigan J.J., Yu K., Ferrari J.D., Jeffrey K.L. (2024). mRNA-delivery of IDO1 suppresses T cell-mediated autoimmunity. Cell Rep. Med..

[89] Krupa A., Kowalska I. (2021). The Kynurenine Pathway-New Linkage between Innate and Adaptive Immunity in Autoimmune Endocrinopathies. Int. J. Mol. Sci..

[90] Myint A.M., Halaris A. (2022). Imbalances in Kynurenines as Potential Biomarkers in the Diagnosis and Treatment of Psychiatric Disorders. Front. Psychiatry.

[91] Marx W., McGuinness A.J., Rocks T., Ruusunen A., Cleminson J., Walker A.J., Gomes-da-Costa S., Lane M., Sanches M., Diaz A.P. (2021). The kynurenine pathway in major depressive disorder, bipolar disorder, and schizophrenia: A meta-analysis of 101 studies. Mol. Psychiatry.

[92] Jasionowska J., Galecki P., Kalinka E., Skiba A., Szemraj J., Turska E., Talarowska M. (2024). Level of selected exponents of the kynurenine pathway in patients diagnosed with depression and selected cancers. J. Psychiatr. Res..

[93] Muthukumar S., Darden J., Crowley J., Witcher M., Kiser J. (2022). A Comparison of PET Tracers in Recurrent High-Grade Gliomas: A Systematic Review. Int. J. Mol. Sci..

[94] Van Binnebeek S., Karges W., Mottaghy F.M. (2011). Functional imaging of neuroendocrine tumors. Methods Mol. Biol..

[95] Toumpanakis C., Kim M.K., Rinke A., Bergestuen D.S., Thirlwell C., Khan M.S., Salazar R., Oberg K. (2014). Combination of cross-sectional and molecular imaging studies in the localization of gastroenteropancreatic neuroendocrine tumors. Neuroendocrinology.

[96] Galldiks N., Langen K.J., Albert N.L., Law I., Kim M.M., Villanueva-Meyer J.E., Soffietti R., Wen P.Y., Weller M., Tonn J.C. (2022). Investigational PET tracers in neuro-oncology-What’s on the horizon? A report of the PET/RANO group. Neuro Oncol..

[97] Zhu A., Lee D., Shim H. (2011). Metabolic positron emission tomography imaging in cancer detection and therapy response. Semin. Oncol..

[98] Horky L.L., Treves S.T. (2011). PET and SPECT in brain tumors and epilepsy. Neurosurg. Clin. N. Am..

[99] Huang X., Pan Z., Doligalski M.L., Xiao X., Ruiz E., Budzevich M.M., Tian H. (2017). Evaluation of radiofluorinated carboximidamides as potential IDO-targeted PET tracers for cancer imaging. Oncotarget.

[100] Oldan J.D., Giglio B.C., Smith E., Zhao W., Bouchard D.M., Ivanovic M., Lee Y.Z., Collichio F.A., Meyers M.O., Wallack D.E. (2023). Increased tryptophan, but not increased glucose metabolism, predict resistance of pembrolizumab in stage III/IV melanoma. Oncoimmunology.

[101] Xie L., Maeda J., Kumata K., Yui J., Zhang Y., Hatori A., Nengaki N., Wakizaka H., Fujinaga M., Yamasaki T. (2015). Development of 1-N-(11)C-Methyl-L- and -D-Tryptophan for pharmacokinetic imaging of the immune checkpoint inhibitor 1-Methyl-Tryptophan. Sci. Rep..

[102] Jarvis L. (2018). Incyte drug failure casts shadow on IDO class. Chem. Eng. News.

[103] He S., Tang G., Hu K., Wang S., Huang T., Liang X. (2016). Preclinical evaluation of 5-([11C]-methyloxy)-L-tryptophan as a potential PET molecular imaging probe. Nucl. Med. Commun..

[104] Kilbourn M.R. (2017). Small Molecule PET Tracers for Transporter Imaging. Semin. Nucl. Med..

[105] Yue X., Xin Y., Zhang S., Nikam R., Kandula V., Choudhary A.K., Chugani H.T., Chugani D.C. (2020). Automated production of 1-(2-[(18)F]fluoroethyl)-l-tryptophan for imaging of tryptophan metabolism. Appl. Radiat. Isot..

[106] Kolks N., Neumaier F., Neumaier B., Zlatopolskiy B.D. (2023). Preparation of N(In)-Methyl-6-[(18)F]fluoro- and 5-Hydroxy-7-[(18)F]fluorotryptophans as Candidate PET-Tracers for Pathway-Specific Visualization of Tryptophan Metabolism. Int. J. Mol. Sci..

[107] Marti-Climent J.M., Prieto E., Moran V., Sancho L., Rodriguez-Fraile M., Arbizu J., Garcia-Velloso M.J., Richter J.A. (2017). Effective dose estimation for oncological and neurological PET/CT procedures. EJNMMI Res..

[108] Martin O., Schaarschmidt B.M., Kirchner J., Suntharalingam S., Grueneisen J., Demircioglu A., Heusch P., Quick H.H., Forsting M., Antoch G. (2020). PET/MRI Versus PET/CT for Whole-Body Staging: Results from a Single-Center Observational Study on 1,003 Sequential Examinations. J. Nucl. Med..

[109] Qiao Z., Mardon K., Stimson D.H.R., Migotto M.A., Reutens D.C., Bhalla R. (2020). Synthesis and evaluation of 6-[18F]fluoro-3-(pyridin-3-yl)-1H-indole as potential PET tracer for targeting tryptophane 2, 3-dioxygenase (TDO). Nucl. Med. Biol..

